# Antenatal care use in Ethiopia: a spatial and multilevel analysis

**DOI:** 10.1186/s12884-019-2550-x

**Published:** 2019-11-01

**Authors:** Teketo Kassaw Tegegne, Catherine Chojenta, Theodros Getachew, Roger Smith, Deborah Loxton

**Affiliations:** 1 Department of Public Health, College of Health Sciences, Debre Markos University, Debre Markos, Ethiopia; 2 Research Centre for Generational Health and Ageing, Hunter Medical Research Institute, School of Medicine and Public Health, University of Newcastle, Newcastle, New South Wales Australia; 3 The Australian College of Health Informatics, Sydney, New South Wales Australia; 4 Health System and Reproductive Health Research Directorate, Ethiopian Public Health Institute, Addis Ababa, Ethiopia; 5 Mothers and Babies Research Centre, Hunter Medical Research Institute, School of Medicine and Public Health, University of Newcastle, Newcastle, New South Wales Australia

**Keywords:** Antenatal care, Prenatal care, Spatial variations

## Abstract

**Background:**

Accessibility and utilization of antenatal care (ANC) service varies depending on different geographical locations, sociodemographic characteristics, political and other factors. A geographically linked data analysis using population and health facility data is valuable to map ANC use, and identify inequalities in service access and provision. Thus, this study aimed to assess the spatial patterns of ANC use, and to identify associated factors among pregnant women in Ethiopia.

**Method:**

A secondary data analysis of the 2016 Ethiopia Demographic and Health Survey linked with the 2014 Ethiopian Service Provision Assessment was conducted. A multilevel analysis was carried out using the SAS GLIMMIX procedure. Furthermore, hot spot analysis and spatial regressions were carried out to identify the hot spot areas of and factors associated with the spatial variations in ANC use using ArcGIS and R softwares.

**Results:**

A one-unit increase in the mean score of ANC service availability in a typical region was associated with a five-fold increase in the odds of having more ANC visits. Moreover, every one-kilometre increase in distance to the nearest ANC facility in a typical region was negatively associated with having at least four ANC visits. Twenty-five percent of the variability in having at least four ANC visits was accounted for by region of living. The spatial analysis found that the Southern Nations, Nationalities and Peoples region had high clusters of at least four ANC visits. Furthermore, the coefficients of having the first ANC visit during the first trimester were estimated to have spatial variations in the use of at least four ANC visits.

**Conclusion:**

There were significant variations in the use of ANC services across the different regions of Ethiopia. Region of living and distance were key drivers of ANC use underscoring the need for increased ANC availability, particularly in the cold spot regions.

## Background

Antenatal care (ANC) is a maternal healthcare service provided by skilled healthcare professionals to pregnant women and adolescent girls. It is provided throughout pregnancy to ensure the best health outcomes for both the mother and the newborn. The care service includes the following components: risk identification, prevention and management of pregnancy-related or concurrent diseases, and health education and health promotion [1]. The World Health Organization (WHO) recommends Midwife-led continuity of care models throughout pregnancy, delivery and postnatal period. Antenatal home visits are also recommended to improve antenatal care utilization and perinatal health outcomes [1].

Antenatal care has the potential to reduce maternal and child morbidity and/or mortality and to improve newborn health [2–6]. For instance, in a systematic review and meta-analysis, it was found that ANC visit was significantly associated with a 34% reduction in neonatal mortality [5]. In a cohort study carried out in Ethiopia, it was reported that having four or more ANC visits was significantly associated with 81.2, 61.3, 52.4 and 46.5% reduction in postpartum haemorrhage, early neonatal death, preterm labour and low-birth weight, respectively [6]. Inadequate antenatal care visits, or late visits, or fewer than the recommended number of visits have been related to poor pregnancy outcomes [7]. A lack of relevant and high quality antenatal care services is a major concern in sub-Saharan Africa [8].

In the case of uncomplicated pregnancies, the 2002 Focused Antenatal Care model of the WHO recommended at least four antenatal care visits; the first visit to take place before 16 weeks of gestation [9]. However, this model has now been superseded by the 2016 WHO ANC model; where a minimum of eight ANC contacts is recommended. The word “visit” in the previous model has now changed to “contact” to indicate an active interaction between a pregnant woman and a health-care provider. The first contact should take place in the first trimester; that is, within the first 12 weeks of gestation. The other recommended contacts are two contacts in the second trimester (at 20 and 26 weeks) and five contacts in the third trimester (at 30, 34, 36, 38 and 40 weeks of gestation) [1].

In the developing regions of the world, according to the United Nations report, over the 25 years period (1990 to 2014), there was slow progress in the use of four or more antenatal care visits [10]. In 2014, on average, only 52% of pregnant women in the developing regions had received a minimum of four antenatal care visits, which was a 17% increase from 1990. It was reported that 36% of pregnant women in Southern Asia and 49% in sub-Saharan Africa had received four or more antenatal care visits in 2014 [10]. In Ethiopia, the rates of women using antenatal care at least once increased from 27 to 62% from 2000 to 2016 [11–14]. In 2016, 31.8% of Ethiopian women received four or more ANC visits [14] and the median timing of the first antenatal care visit was 5.2 months [13]. About 63 and 89% of women from urban areas and Addis Ababa respectively had four or more antenatal care visits as compared to 27% from rural areas and those from the Somali region (11.8%).

Both the demand and supply side factors are important in determining health service use. However, the majority of previous studies were mainly focused on the demand side factors of health service use. For instance, most studies identified the demand side factors of antenatal care use [15–20]. Amongst the demand side factors, women’s education [16, 21, 22], husband’s occupation [21], socioeconomic status [16], and place of residence [16, 17] were significantly associated with the use of antenatal care service. In most studies, the supply side factors of ANC use have been overlooked. Understaffed health facilities [23] and distant ANC facilities [23, 24] were negatively associated with the use of antenatal care services. The most important supply side factors, such as health facilities general service readiness, availability of ANC services and facilities readiness to provide ANC service [25, 26] were not addressed. Due to the increasing availability of georeferenced health facility and population data, it is important to link these data sets and identify both the demand and supply side factors of ANC use.

So far, in Ethiopia, four Demographic and Health Surveys (DHS) were conducted, but the Service Provision Assessment (SPA) survey was the first to be carried out in the country. The DHS survey provides detail information about population characteristics including their health service use history [27]. On the other hand, the SPA survey provides information about healthcare services available at each health facility and facility’s readiness to provide a particular service [28]. Using the capability of geographic information system (GIS), a linked analysis of population and health facility data has enormous importance for investigating the links between population healthcare needs and uses, and the health service environment [29]. Despite this importance, these two datasets have not been used in Ethiopia. Therefore, this study aimed to assess the spatial variations in the use of antenatal care services among women who gave birth in a 5 year period in Ethiopia. Furthermore, it aimed to identify the potential factors associated with the use of antenatal care services throughout the country using national population and health facility data.

## Methods

### Data sources

This study used two data sources: the Ethiopian Demographic and Health Survey (EDHS) and the Ethiopian Service Provision Assessment Plus (ESPA+) survey data sets.

### Population data

The main source of the population data were the 2016 EDHS. The survey used a stratified sampling procedures. Data on demographic characteristics and population health service use, such as antenatal care were collected [14]. Geographic coordinates of each survey cluster were also collected using Global Positioning System (GPS) receivers. Survey clusters are basically called Enumeration Areas (EAs). An EA is a geographical location that has an average of 181 households [14]. The GPS reading was collected at the centre of each cluster. For the purpose of insuring respondents’ confidentiality, GPS latitude/longitude positions for all surveys were randomly displaced before public release. The maximum displacement was two kilometres for urban clusters and five kilometres for 99% of rural clusters. One percent of rural clusters were displaced a maximum of 10 km. The displacement was restricted within the country’s second administrative level [30].

The survey collected data on all women aged 15–49 years in the 645 clusters. This study used 6954 women who gave birth in the 5 years preceding the survey in the 622 clusters. A total of 239 women who gave birth in the 5 years preceding the survey in the other 23 clusters were excluded from the analysis since they had no geographic information.

### Health facility data

The main source of the health facility data were the 2014 ESPA+ survey. This survey data was obtained from the Ethiopian Public Health Institute (EPHI). Ethics application was submitted to EPHI before obtaining the dataset. It is the main source of data on the availability of health services, such as antenatal and delivery care services [31]. Health facilities were selected using a combination of census and simple random sampling techniques [31]. In this analysis, amongst the 1165 interviewed health facilities [32], 919 facilities which reported providing antenatal care were included.

### Data linking method

In this analysis, administrative boundary link was used for linking sampled health facility data with the population data [32]. This is the method of choice for linking sampled health facilities data with population data [29, 32, 33]. Details of this method are discussed elsewhere [32]. The administrative boundaries of Ethiopia were obtained from Natural Earth [34].

### Health service environment and measurements

Four antenatal care service environment variable scores were created. These were average distance to the nearest antenatal care facilities, antenatal care service availability score, readiness to provide antenatal care services score, and a general health facilities readiness score. All service availability and readiness scores were computed for the nearest health facilities. The antenatal care indices were created using the World Health Organization’s ‘Service Availability and Readiness Indicators’ [25, 26]. Details of computing these scores are discussed elsewhere [32].

After linking DHS clusters with SPA facilities, average straight-line distance to the nearest antenatal care health facility was calculated [32]. First, the distance from each cluster to every ANC facility within the administrative boundaries (that is within regions) was calculated. Second, the nearest facility was identified and selected for each cluster. Lastly, using the identified nearest facility, average distance was calculated per region and used in this paper.

With regard to general service readiness score, out of the 12 WHO general service readiness variables [25, 26], the principal component analysis gave nine general service dimensions [32]. The SCORE procedure in SAS was also used to compute the average general service readiness score per region/city administration. These were 24-h staff coverage, communication equipment, clean water source, power supply, management meetings, client opinion/feedback, quality assurance, emergency transport, and client latrine. The first two principal components (health facility management system and infrastructure) were used to compute two general service readiness scores.

For those health facilities reported as providing antenatal care services, indices of antenatal care availability and readiness were created. One antenatal care availability score (antenatal care supplements) was created using four variables [32]. Similarly, two antenatal care readiness scores (readiness to provide diagnostic services and skilled care) were created using six variables [32]. Along with the principal component analysis [32], the SCORE procedure in SAS was used to compute average ANC service availability and readiness scores per region/city administration.

Antenatal care use was defined based on the number of antenatal visits a woman had for her most recent birth in the 5 years preceding the survey. A woman was grouped into either of the three categories: 1) had no ANC visit, 2) one to three ANC visits or 3) four or more ANC (ANC4+) visits. The DHS survey collected data on pregnancy status of the last birth as wanted, wanted later and unwanted birth. The survey asked a woman whether the child was wanted at the time of pregnancy, or whether the child was wanted but later, or whether the child was not wanted at all. In this paper, pregnancy status was classified as wanted if a woman wanted it at the time of pregnancy and unwanted if a woman wanted it later or was not wanted at all.

### Statistical analysis

#### Hierarchical generalized linear model

A two level multilevel regression analysis was carried out after linking women in the respective cluster to the health facility variables. This study had ordinal polytomous outcome with three groups of ANC visits: 1) No ANC visit, 2) one-to-three ANC visits and 3) four or more ANC (ANC4+) visits. We were interested in the probability of being at or above zero level of ANC visit and the influence of individual and region characteristics on this probability for each category. Multiple logits are simultaneously estimated (M-1 logits, where M is the number of response categories) when analysing polytomous outcomes. Therefore, in this study with three categories of response, there will be two logits and their corresponding intercepts will be simultaneously estimated, each of them indicating the probability of responding in or above a particular category.

The equations necessary for estimating these two level models are presented below [35].

$$ {Y}_{1 ij}=\mathit{\log}\left[\frac{P\left({R}_{ij}\le 1\right)}{1-P\left({R}_{ij}\le 1\right)}\right]={\gamma}_{00}+{\gamma}_{10}{x}_{ij}+{\gamma}_{01}{W}_j+{\mu}_{0j}+{\mu}_{1j}{x}_{ij} $$

$$ {Y}_{2 ij}=\mathit{\log}\left[\frac{P\left({R}_{ij}\le 2\right)}{1-P\left({R}_{ij}\le 2\right)}\right]={\gamma}_{00}+{\gamma}_{10}{x}_{ij}+{\gamma}_{01}{W}_j+\delta +{\mu}_{0j}+{\mu}_{1j}{x}_{ij} $$

*Where* ***Y***_***ij***_ *represents the log odds of being at or above zero level of ANC visit for woman* ***i*** *in region* ***j****. More specifically, Y*_1*ij*_ *corresponds to the log odds of being at or above the highest ANC visit (*i.e.*, ANC4+) for woman* ***i*** *in region* ***j, P***(***R***_***ij***_ ***≤*** **1**) *represents the probability of responding at or above this highest ANC visit, and* **1** ***− P***(***R***_***ij***_ ***≤*** **1**) *corresponds to the probability of responding below this highest ANC visit for woman* ***i*** *in region* ***j. γ***_**00**_ *provides the log odds of being at or above that ANC visit in a typical region,* ***W***_***j***_ *is a region-level predictor for region* ***j****,* ***γ***_**01**_ *is the slope associated with this predictor,* ***μ***_**0*j***_ *is the level-2 error term representing a unique effect associated with region* ***j, γ***_**10**_ *is the average effect of the individual-level predictor,* ***X***_***ij***_ *is an individual-level predictor for woman* ***i*** *in region* ***j****, and* ***μ***_**1*j***_ *is a random slope for a level-1 predictor variable* ***X***_***ij***_*, which allows the relationship between the individual-level predictor (****X***_***ij***_*) and the outcome (****Y***_**1*ij***_*) to vary across level-2 units. In addition,* ***Y***_**2*ij***_ *represents the log odds of being at or above the next level of ANC visit (*i.e.*, one-to-three ANC visits) for woman* ***i*** *in region* ***j. Y***_**2*ij***_ *also include an extra term,* ***(δ****), representing the difference between the intercepts corresponding to this category and the preceding one. This model gives log odds when fitted to data.*

For the ease of interpretation, the log odds can be converted into probabilities. The predicted probability of the event of interest (being at or above zero level of ANC visit) can be calculated using the following formula as discussed elsewhere [35].

$$ Predicted\ Probability\ {\theta}_{Mij}=\frac{e^{Y_{Mij}}}{1+{e}^{Y_{Mij}}} $$

This is a simple conversion of log odds of an event of interest to a probability of an event of interest. In this expression, ***θ***_***Mij***_ is the probability of the event (being at or above zero level of ANC visit), $\mathbf{1}-{\boldsymbol{e}}^{{\boldsymbol{Y}}_{\boldsymbol{Mij}}}$ is the corresponding probability of being below a given ANC visit, Y_Mij_ represents the log odds of the event of interest that is the log odds of pregnant woman ***i*** in the ***j***^***th***^ region being at or above ***M***^***th***^ level of ANC visit.

Ordinal polytomous data can be modelled using multinomial distribution with cumulative logit link function to compute the cumulative odds ratio for each category of response variable [36]. The GLIMMIX procedure in SAS was used to estimate the hierarchical generalized linear model [35]. The GLIMMIX procedure fits two kinds of models to multinomial data. For ordinal data, models with cumulative link functions apply, whereas generalized logit models fit for nominal data. Using cumulative logit link function reduces model size and memory requirements as compared to using generalized logit link function. The multilevel multinomial logistic regression model was carried out to predict the probability of being at or above zero level of ANC visit using different individual and region level variables. Since the outcome variable is ordinal, the cumulative logit (CLOGIT) link function was used.

Four model building process steps were followed. The Laplace estimation in the GLIMMIX procedure was used to estimate the best-fitting model [35]. The model building process was started with the empty, unconditional model with no predictors. This model was used to calculate the intra-class correlation coefficient (ICC) [35]. The ICC estimate tells how much variation in the use of antenatal care exists between regions (level-2 units) [32]. Details of calculating ICC in hierarchical generalized linear models is discussed elsewhere [32, 37]. A more complex models were gradually built by checking improvements in model fit after each model was estimated. The negative 2 log likelihood (−2LL) was used to assess the best fitting model [35].

Model two was built by including level-1 (individual level) variables as fixed effects in the random intercept only model (empty model). The individual level variables included in this model were women’s age, women’s education, women’s occupation, husband’s education, husband’s occupation, household wealth, parity, timing of 1st ANC visit, age at 1st birth, number of living children, nature of recent pregnancy (planned or unplanned) and autonomy in own healthcare decision making. However, only five individual level variables: husbands’ or partners’ education, household wealth, number of living children, autonomy in own personal healthcare decision making and nature of pregnancy were found significant and carried forward. Then, model three was built by adding these individual level variables as random effects in order to determine if their influence on ANC visit varied among regions. Lastly, level-2 (region level) variables were added as fixed effects in the fourth model: urban-rural residence, two general service readiness scores (health facility management system and infrastructure), ANC availability score (ANC supplements), two ANC readiness scores (readiness to provide diagnostic services and skilled care) and average straight-line distance to the nearest ANC health facility.

#### Spatial analysis

The spatial analysis was carried out using ArcGIS 10.6.1 and R version 3.5.1. To produce the flattened map of Ethiopia, the Ethiopian Polyconic Projected Coordinate System was used [32]. Hot spot analysis and spatial regression were carried out to identify spatial clusters and factors associated with spatial variations of antenatal care use, respectively. Since geographic coordinates were collected at cluster level, the unit of spatial analyses were DHS clusters.

The hot spot analysis followed three procedures as discussed elsewhere [32]. These were the Global Moran’s I statistic, Incremental Spatial Autocorrelation and the Getis-Ord Gi* statistic. The Global Moran’s I statistic is a global measure of spatial autocorrelation [38]. The Incremental Spatial Autocorrelation was used to determine the scale, which is the critical distance at which there is maximum clustering [32]. The average distance at which a feature has at least one neighbour (15 km) was calculated using *Calculate Distance Band from Neighbour Count* in the Spatial Statistics tools toolbox in ArcGIS. The maximum distance at which clustering of at least four antenatal care visits peaked was at 104 km with a z-score of 4.02. The Getis-Ord Gi* statistic used this maximum distance to identify statistically significant spatial clusters of hot spots (areas of high antenatal care use rates and cold spots (low antenatal care use areas). A False Discovery Rate (FDR) correction method was applied to account for multiple and spatial dependence tests in Local Statistics of Spatial Association [32, 39]. Statistical significance was determined based on the z-scores and *p*-values returned while running hot spot analysis [32].

In addition to analysing the spatial patterns (hot spots), spatial regression was carried out to identify key factors behind the observed spatial patterns of ANC use. Moran’s eigenvector-based spatial regression analysis was carried out using the *spmoran* package in R. Ordinary least square (OLS) regression models are frequently used to analyse and model spatial data. However, regression models applied to spatial data are frequently associated with spatially autocorrelated residuals. An eigenvector spatial filtering (ESF) regression models effectively removes these spatially autocorrelated residuals [40]. ESF reduces spatial misclassification errors, and it increases strength of model fit, normality of model residuals and homoscedasticity of model residuals [41]. In this analysis, a random effects eigenvector spatial filtering (RE-ESF) regression model that is the extension of ESF was used. RE-ESF estimates of regression coefficients and their standard errors are more accurate and reliable than ESF [42]. All the variables considered in the multilevel analysis were included in the spatial regression analysis.

Furthermore, Moran eigenvector spatially varying coefficient (M-SVC) model, a local form of linear regression was used to model spatially varying relations. The M-SVC model outperforms the geographically weighted regression (GWR) model, which is a standard spatially varying coefficient (SVC) modelling approach, in terms of coefficient estimation accuracy and computational time [43].

## Results

### Sociodemographic characteristics

The mean age of women who gave birth in the 5 years period was 29.27 (standard deviation of ±6.82) years. Approximately 28% of women were within the age range of 25–29 years. Over 59% of the women had no education, while 27.37% had a primary level education. With regard to wealth, 23.45% of the women fell in the richest quintile and 32.56% were grouped in the poorest quintile. About 34 and 45% of the women were followers of the Orthodox Christian and Muslim faith, respectively. Seventy eight percent of the women were from rural areas (Table 1).

**Table 1:** Sociodemographic characteristics of pregnant women in Ethiopia, 2016 (*N* = 6954)

Variable	Frequency	Percentage
Age		
15–19	343	4.93
20–24	1434	20.62
25–29	1957	28.14
30–34	1484	21.34
35–39	1115	16.03
40–44	462	6.64
45–49	159	2.29
Women’s education		
No education	4171	59.98
Primary	1903	27.37
Secondary	567	8.15
Higher	313	4.50
Women’s occupation		
Have no work	5013	72.09
Professional work	905	13.01
Agricultural work	713	10.25
Other	323	4.64
Husbands’/partners’ education		
No education	2997	43.10
Primary	2108	30.31
Secondary	726	10.44
Higher	1123	16.15
Husbands’/partners’ occupation		
Have no work	639	9.19
Professional work	1730	24.88
Agricultural work	3454	49.67
Others	1131	16.26
Head of household		
Someone else	5775	83.05
Herself	1179	16.95
Family size		
1–4	2273	32.69
5–8	3872	55.68
> = 9	809	11.63
Wealth quintile		
Lowest	2264	32.56
Second	1157	16.64
Middle	1007	14.48
Fourth	895	12.87
Highest	1631	23.45
Religion		
Orthodox	2360	33.94
Protestant	1334	19.81
Muslim	3099	44.56
Other	161	2.32
Residence		
Urban	1512	21.74
Rural	5442	78.26

### Women’s obstetric characteristics

Amongst the 6954 women who gave birth 5 years preceding the survey, 2524 (36.30%) had five or more births; and about 30% of the women had more than four living children. The mean age at first childbirth was 19.15 (standard deviation of ±3.74) years. With regard to healthcare decisions, only 16.31% of the women had autonomy to decide on their own healthcare needs (Table 2).

**Table 2:** Obstetric characteristics of pregnant women in Ethiopia, 2016 (*N* = 6954)

Variable		Frequency	Percentage
Parity	1–4	4430	63.70
	> = 5	2524	36.30
Number of living children	0	64	0.92
	1–4	4757	68.41
	> = 5	21.33	30.67
Age at first childbirth	<= 19 years	4250	61.12
	20–24 years	2087	30.01
	> = 25 years	617	8.87
Autonomy in own personal healthcare decision making	Respondent alone	1134	16.31
	Joint decision	4063	58.43
	Husband/partner alone	1757	25.27
Timing of first ANC visits (*n* = 4603)	1st trimester	1786	38.80
	2nd Trimester	2396	52.05
	3rd Trimester	421	9.15
Nature of recent pregnancy	Wanted	5516	79.32
	Unwanted	1438	20.68

### Health facility characteristics

Data were collected from 1165 health facilities nationwide. Amongst these health facilities, 18.73 and 27.75% were hospitals and health centres, respectively. About 68% of the health facilities were managed by the government. With regard to antenatal care service provision, 919 (78.88%) of the health facilities provided antenatal care services. The national average distance from antenatal care health facilities to the 2016 EDHS clusters was 9.95 km. The 2016 EDHS sampled clusters in the Somali region were the longest distance (25.46 km) from antenatal care facilities. Conversely, EDHS clusters in Dire Dawa were 0.99 km from antenatal care facilities (Table 3).

**Table 3:** The average distance from sampled antenatal care providing health facilities to demographic and health survey clusters in Ethiopia, 2016 (*N* = 919)

Region	Health facility type				Average distance (km)	Interquartile range (km)
	Hospitals n (%)	Health Centres n (%)	Health Posts n (%)	Private Clinics n (%)		
Tigray	30 (30.93)	31 (31.96)	23 (23.71)	13 (13.40)	9.41	2.38–14.12
Afar	6 (11.32)	25 (47.17)	18 (33.96)	4 (7.55)	15.31	3.57–20.73
Amhara	26 (17.81)	46 (31.51)	31 (21.23)	43 (29.45)	13.61	7.40–19.01
Oromia	52 (30.77)	50 (29.59)	40 (23.67)	27 (15.98)	14.50	7.46–19.75
Somali	10 (18.52)	24 (44.44)	14 (25.93)	6 (11.11)	25.46	4.76–40.73
Benishangul-Gumuz	2 (3.92)	16 (31.37)	28 (54.90)	5 (9.80)	9.43	3.30–13.30
SNNPR	26 (18.18)	40 (27.97)	38 (26.57)	39 (27.27)	11.64	6.27–14.74
Gambela	1 (2.63)	16 (42.11)	16 (42.11)	5 (13.16)	6.87	0.76–11.41
Harari	4 (10.26)	8 (20.51)	20 (51.28)	7 (17.95)	1.21	0.47–1.67
Addis Ababa	37 (52.11)	18 (25.35)	0	16 (22.54)	1.02	0.48–1.32
Dire Dawa	6 (10.34)	15 (25.86)	31 (53.45)	6 (10.34)	0.99	0.45–1.38
Total	200 (21.76)	289 (31.45)	259 (28.18)	171 (18.61)	10.41	1.38–14.81

The mean of health facilities service availability and readiness scores were different for each region and city administration. For Addis Ababa, the average value of health facility infrastructure was 0.783, while for the Benishangul-Gumuz region the average value was much lower. The Benishangul-Gumuz region health facilities had the lowest mean value of − 0.534. Regarding facilities diagnostic services, health facilities in Dire Dawa had the highest readiness score of 0.540, while Gambela had the lowest mean value of − 0.544. The mean scores of health facility service availability and readiness for each region and city administration is shown in Table 4.

**Table 4:** Health facilities service availability and readiness scores linked to the demographic and health survey clusters in Ethiopia, 2016 (*N* = 919)

Region	Facility management system	Facility infrastructure	ANC supplements	Skilled care	Diagnostic services
Tigray	0.526	0.163	0.297	0.545	0.126
Afar	−0.340	0.035	− 0.105	− 0.052	− 0.213
Amhara	0.115	0.147	−0.046	0.045	−0.143
Oromia	0.025	−0.055	−0.068	0.145	−0.162
Somali	−0.165	−0.623	0.168	−0.270	− 0.116
Benishangul-Gumuz	−0.184	− 0.534	−0.187	0.490	0.206
SNNPR	0.151	−0.166	−0.073	− 0.345	−0.082
Gambela	−0.717	−0.504	− 0.030	−0.074	− 0.544
Harari	−0.437	0.249	−0.687	− 0.333	0.437
Addis Ababa	0.053	0.783	0.292	−0.021	0.268
Dire Dawa	0.013	0.043	0.227	−0.081	0.540

### Antenatal care use

**Fig. 1:**
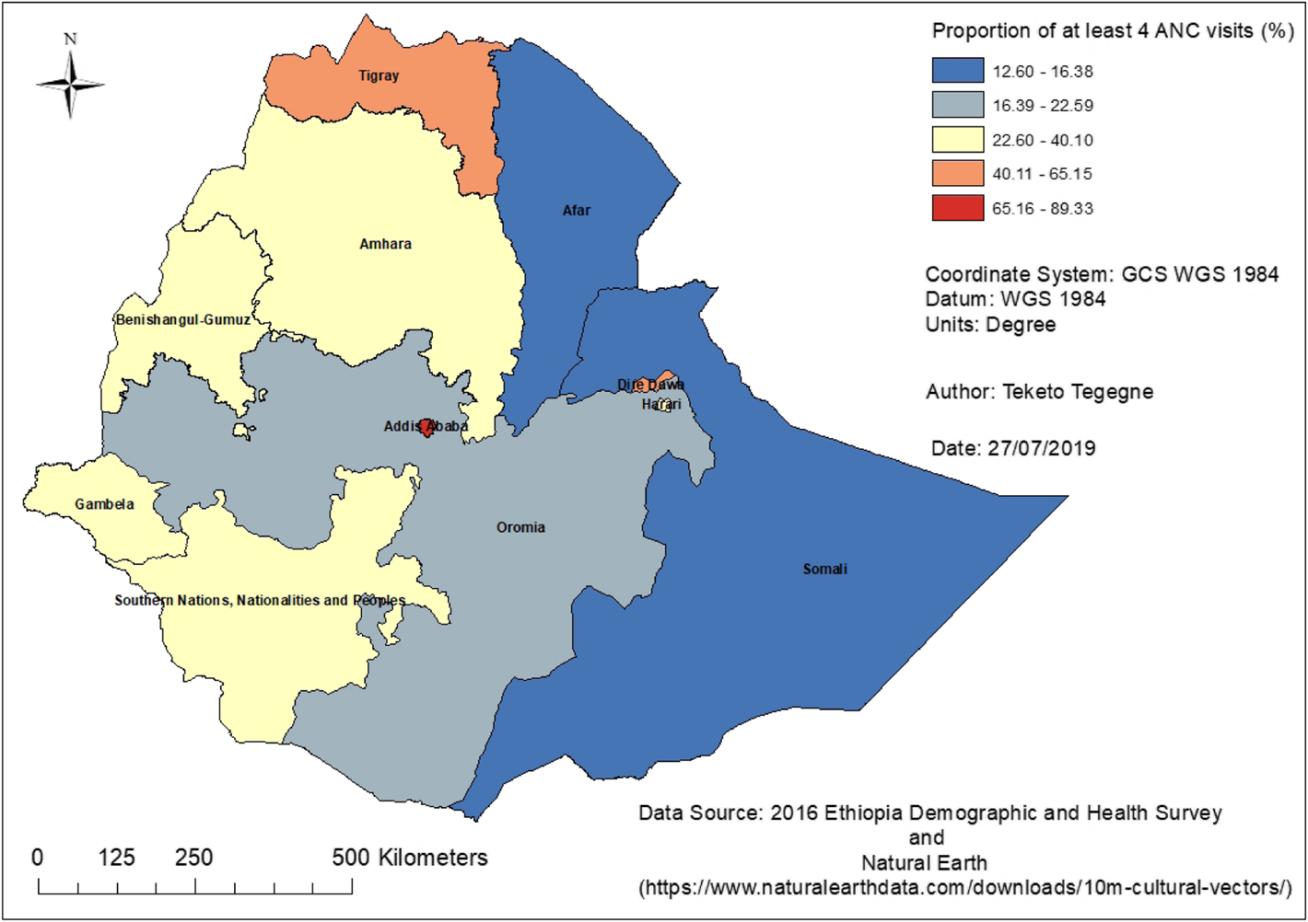
Antenatal care use among pregnant women in Ethiopia, 2016

There were 2351 (33.81%) women who reported having no ANC visits for their last pregnancy. The proportion of women who had at least four ANC visits during their last pregnancy was 36.78% (66.93% urban, 28.41% rural). Utilization of antenatal care services varied across the different regions and city administrations; for instance, the highest number of four or more antenatal care visits were reported in Addis Ababa (89.33%), followed by Dire Dawa (65.15%) and the Tigray Region (55.83%). The map (Fig. 1) shows the regional variations in antenatal care use rates.

### Determinants of antenatal care use

The calculated intra-class correlation coefficient (ICC) was 25.00%. This indicated that 25% of the variability in having one or more ANC visits was accounted for by region, leaving 75% of the variability to be accounted for by the differing characteristics of the women, or other unmeasured factors. The ICC was calculated using an intercept variance (${\sigma}_{\mu 0}^2$) and level-1 residual variance (***ε***_***ij***_**)** as:

$$ ICC=\frac{\sigma_{\mu 0}^2}{\sigma_{\mu 0}^2+\raisebox{1ex}{${\pi}^2$}\!\left/ \!\raisebox{-1ex}{$3$}\right.}=\frac{1.0968}{1.0968+3.29}=0.25 $$

The probability of being at or above ANC4+ visits for a given woman with average background characteristics (with no covariate in the model) was 0.3945. This is the probability of having at least four ANC visits in a typical region.

$$ P\left( being\  at\  or\ above\  ANC4+ visits\right)=\frac{e^{-0.4284}}{1+{e}^{-0.4284}}=\frac{0.6516}{1+0.6516}=0.3945 $$

Similarly, the probability of having at least one-to-three ANC visits or more was 0.7263.

$$ P\left( being\  at\  or\ above\  one- to- three\  ANC\  visits\right)=\frac{e^{0.9762}}{1+{e}^{0.9762}}=\frac{2.6544}{1+2.6544}=0.7263 $$

This is the cumulative probability of being at or above one-to-three ANC visit. In order to obtain the exact probability of being at a given level, we have to subtract the adjacent probabilities. For example, the predicted probability of having ANC4+ visit was 0.3945, one-to-three ANC visit was (0.7263 − 0.3945 = 0.3318) and also the probability of having no ANC visit was 1 − 0.7263 = 0.2737.

Women’s autonomy in own healthcare decision, husbands’ / partners’ education, household wealth, number of living children a woman had, and nature of pregnancy were strong individual-level predictors of having more ANC visits (i.e., ANC4+ visits) among pregnant women. A woman whose husband/partner made decisions on her own healthcare was 24% less likely to have at least four ANC visits as compared to a woman who had autonomy to make decisions. A woman whose husband attained a primary level of education was 53% more likely to have more ANC visits as compared to those whose husband had no education. The odds of having at least four ANC visits increased with increasing wealth quintile. Therefore, women who were in the highest quintile were 3.48 times more likely to have more ANC visits as compared to those in the lowest quintile. Women whose pregnancy was unwanted were 20% less likely to have more ANC visits relative to their counterparts with a wanted pregnancy. Moreover, a one-child increase in the number of children a woman had was associated with a 7 % decrease in the use of more ANC visits (Table 5).

**Table 5:** Factors associated with being at or above one-to-three ANC visits among pregnant women in Ethiopia (*N* = 6954)

Predictors		N	Adjusted odds ratio (95% CI)
			Being at or above one-to-three ANC visits
Level-1 predictor variables			
Husbands’ or partners’ education	No education	4171	1
	Primary	1903	**1.526 (1.322, 1.763)**
	Secondary	567	**2.023 (1.650, 2.480)**
	Higher	313	**1.72 (1.457, 2.107)**
Wealth quintile	Lowest	2264	1
	Second	1157	**2.004 (1.591, 2.524)**
	Middle	1007	**2.273 (1.786, 2.892)**
	Fourth	895	**3.107 (2.421, 3.987)**
	Highest	1631	**3.483 (2.650, 4.578)**
Number of living children			**0.929 (0.897, 0.962)**
Autonomy in own personal healthcare decision making	Respondent alone	1134	1
	Joint decision	4063	0.995 (0.844, 1.174)
	Husband/partner alone	1757	**0.763 (0.634, 0.919)**
Nature of pregnancy	Wanted	5516	1
	Unwanted	1438	**0.803 (0.692, 0.930)**
Level-2 predictor variables			
Residence	Urban	1512	1
	Rural	5442	**0.525 (0.415, 0.663)**
General service readiness	Health facility management system		1.016 (0.344, 3.003)
	Health facility infrastructure		0.957 (0.361, 2.535)
Antenatal care service availability	ANC supplements		**4.909 (1.548, 15.569)**
Antenatal care service readiness	Skilled care		1.564 (0.617, 3.962)
	Diagnostic service		1.951 (0.768, 4.957)
Average distance to the nearest health facility			**0.982 (0.968, 0.995)**
Random effects (Error variance)			
Var (Husbands’ or partners’ education)			0.005 (0.001, 16.324)
Var (Wealth quintile)			0.032 (0.015, 0.115)
Var (Number of living children)			0.002 (0.001, 0.018)
Var (Nature of last pregnancy)			0.001 (0.000, 2.400)
Var (Autonomy on her own healthcare decision)			0.007 (0.001, 2.250)
Var (constant) - level-2 variance			0.024 (0.007, 0.692)
Rho – Intra-class correlation			0.0073
Fit statistics (−2 Log Likelihood)			12,857.23

Bold entries have signficant values

At the regional level (level 2), three variables were significantly associated with having more ANC visits (ANC4+ visits). Pregnant women who were living in rural areas were 47% less likely to have more ANC visits as compared to urban women. A one-unit increase in the mean score of antenatal care service availability in a typical region was associated with a five-fold increase in the odds of having more ANC visits. Every one-kilometre increase in distance to the nearest ANC providing facilities in a typical region was negatively associated with having at least four ANC visits (Table 5).

Finally, the majority of the between region variance was explained by this model: the between region variation in using more ANC visits decreased from 1.0968 to 0.0242, which is a 97.74% reduction in the unexplained variance between region antenatal care utilization. However, region level random effects are significant; the intra-class correlation is still 1%. This indicated that even after controlling for individual and regional level factors, there is still a considerable region level clustering of ANC use. The between region variance of slopes indicated that the following two variables varied significantly across regions: household wealth and number of living children (Table 5).

### Hot spots of antenatal care use

**Fig. 2:**
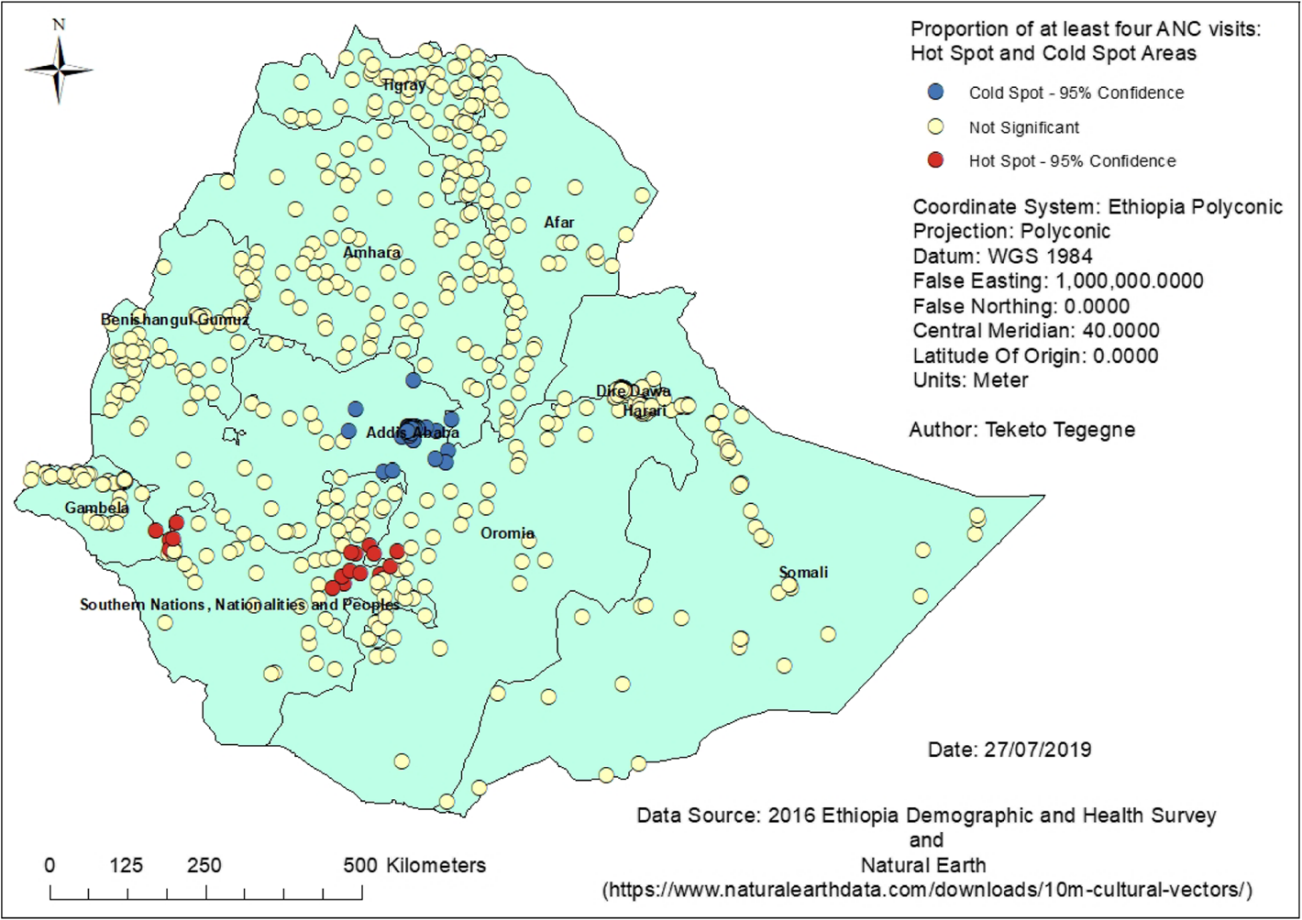
Clusters of at least four ANC visits in Ethiopia, 2016

**Fig. 3:**
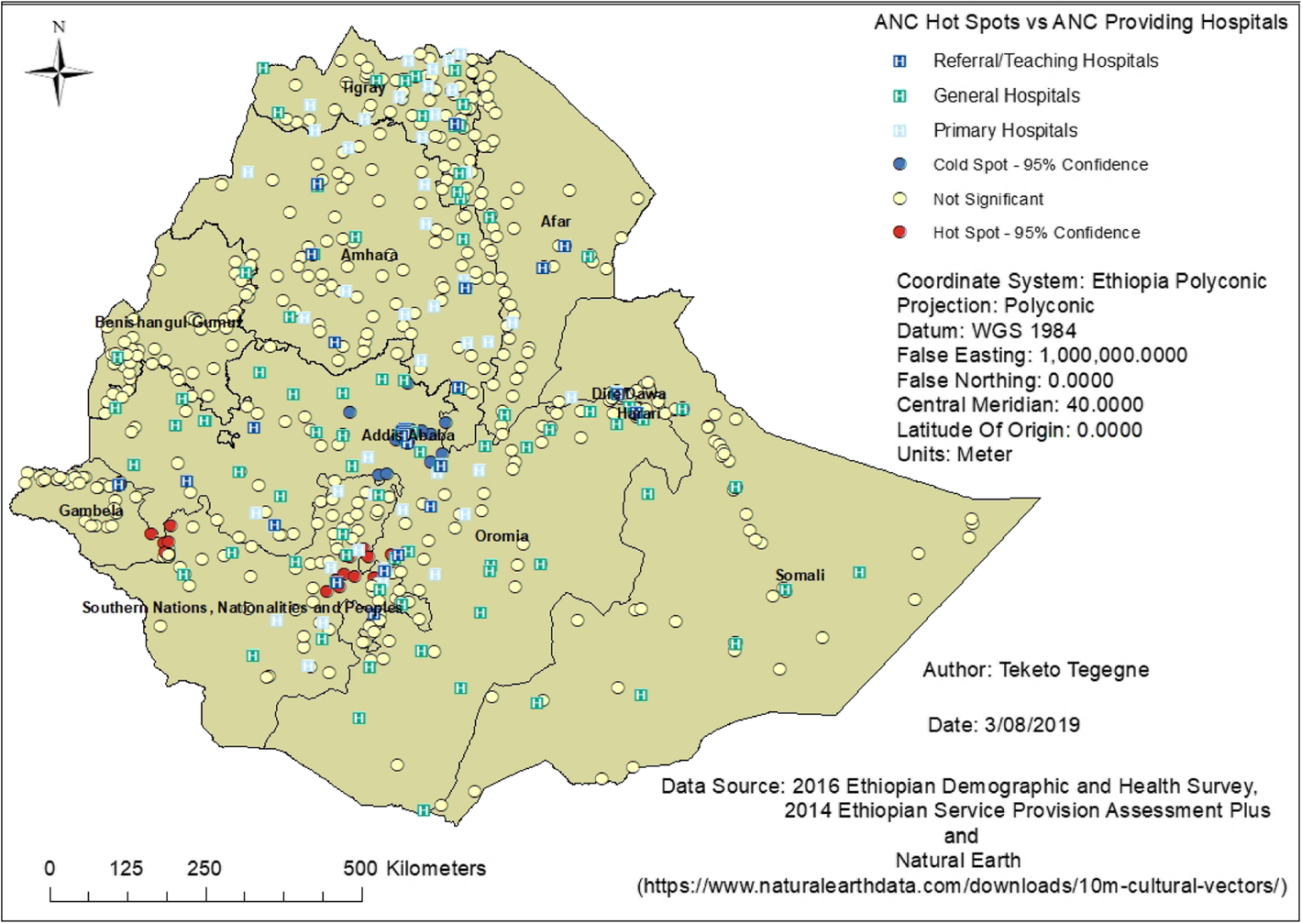
Hot spots of at least four ANC visits vs ANC providing Hospitals in Ethiopia, 2016

There is strong evidence to support spatial clustering in utilization of at least four ANC visits among pregnant women in Ethiopia (Global Moran’s I = 0.18, z-score = 6.11, P-value < 0.0001). Most of the hot spot areas (high ANC rates) were located in the Southern Nations, Nationalities and Peoples region (SNNPR), followed by some parts of the Gambela and Oromia regions. Conversely, the majority of the cold spot areas (low ANC rates) were located in Addis Ababa, followed by some parts of the Oromia region. This clustering was supported by the Getis-Ord Gi* statistic when conducting the spatial analysis (Fig. 2). Furthermore, the identified ANC hot spots were located closest to referral/teaching hospitals. This was observed on the layered map showing hot spots and ANC providing hospitals (Fig. 3).

### Determinants of spatial variations in antenatal care use

In the spatial regression analysis, it was found that household wealth, having first ANC visit during the first trimester, women’s age, parity, availability of ANC supplements, facilities readiness to provide skilled care and distance to ANC providing facilities were associated with the spatial variations of at least four ANC visits. Parity and distance to ANC facilities were negatively associated with the spatial variations in the use of ANC visits (Table 6). About 74% of the spatial variability in the outcome variable was explained by the regression model (adjusted R^2^ = 0.743).

**Table 6:** Factors associated with the spatial variations of at least four ANC visits in Ethiopia, 2016

Variables	Estimate	Standard error	*t*-value	*p*-value
Women’s higher education	0.097	0.057	1.710	0.088
Highest wealth quintile	0.142	0.028	5.117	0.000
ANC visit at first trimester	0.452	0.038	11.857	0.000
Women’s age	0.011	0.003	3.309	0.001
Parity	−0.047	0.011	−4.327	0.000
Availability of ANC supplements	0.299	0.060	4.975	0.000
Facilities readiness to provide skilled care	0.190	0.064	2.948	0.003
Distance to ANC facilities	−0.002	0.001	−2.200	0.028

**Fig. 4:**
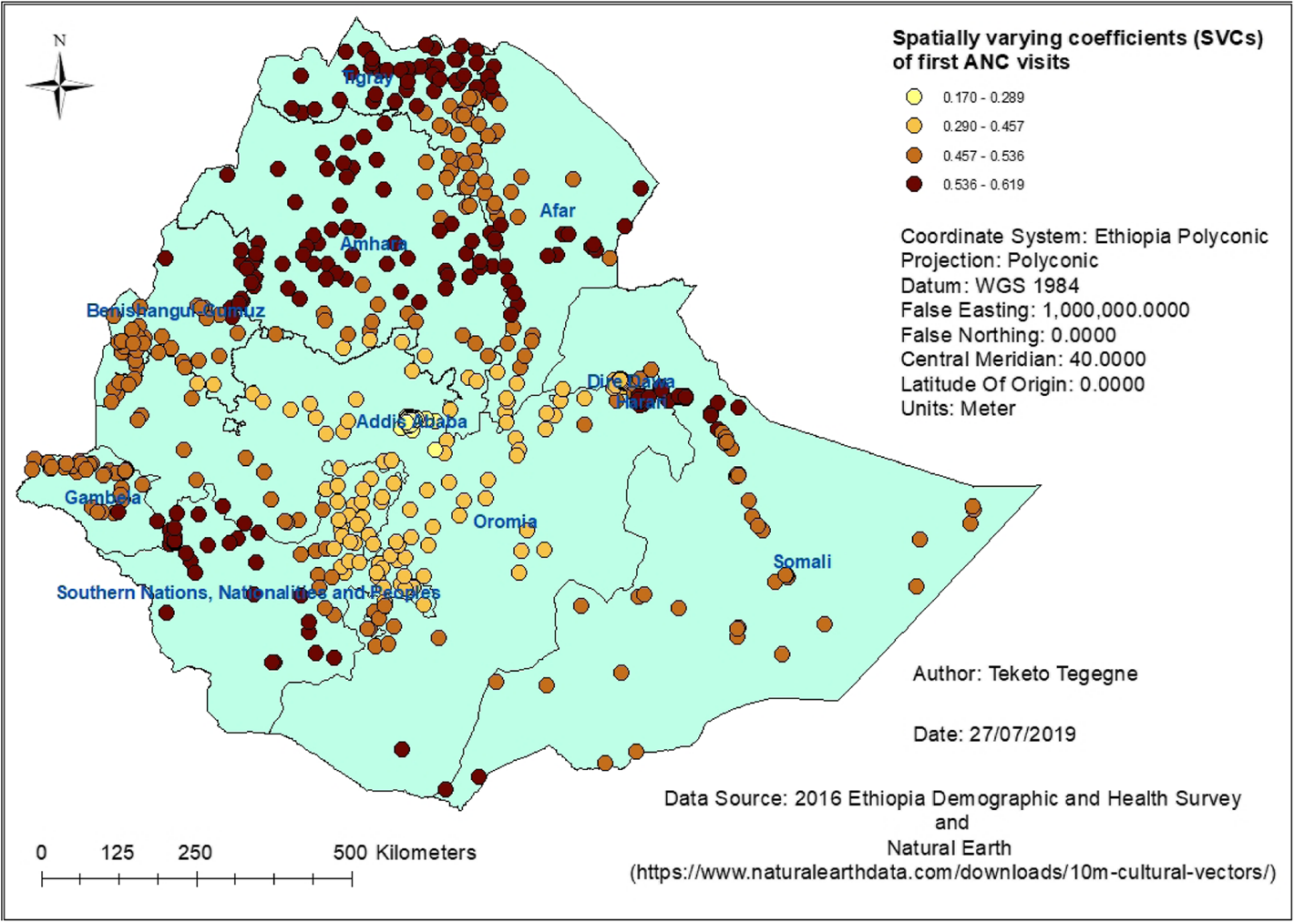
Spatial variations of at least four ANC visits with spatially varying coefficients of first ANC visit in Ethiopia, 2016

In the M-SVC regression model, it was found that the relationship between having at least four ANC visits and having first ANC visit during the first trimester was varying across the geographic space – across clusters (Fig. 4). ANC visit was found to be a spatial problem in Ethiopia. Having first ANC visit during the first trimester was positively associated with having at least four ANC visits across the country. For example, in most of the clusters in the Amhara and Tigray regions, it was found that first ANC visits were stronger predictors of at least four ANC visits. However, coefficients of the other variables were estimated constant. The M-SVC model has explained 75.82% of the spatial variations of having at least four ANC visits.

## Discussion

This study aimed to assess the spatial variations in the use of ANC in Ethiopia. Furthermore, it aimed to identify factors associated with ANC use throughout the country, using the national population and health facility data. This is the first study to provide a comprehensive assessment of ANC use in Ethiopia by region of living, service type and demographics. ANC visit was found to be a spatial problem in Ethiopia. It was also found that women’s ANC visit was significantly associated with different individual and region level factors.

In Ethiopia, the proportion of at least four ANC visits was 36.78%; the highest proportion was reported in urban areas (66.93% vs 28.41%). More than half of antenatal care visits were started during the second trimester of pregnancy, this was far below the WHO recommendation of having at least four ANC visits [9] started during the first trimester of pregnancy [1, 9]. Nevertheless, in 2016, more women had at least four ANC visits as compared to the results of the previous three DHS surveys [11–13]. Similarly, the proportion of women who received ANC in the first trimester was higher than the 2000, 2005 and 2011 DHS survey findings [11–13]. Despite these improvements, this figure is lower than the 2014 United Nations report where 52% of pregnant women in developing regions and 49% in sub-Saharan Africa had a minimum of four ANC visits [10]. There are also variations in ANC visits across the different regions in the country. The highest proportions, more than 50% with at least four ANC visits, were reported in the Addis Ababa and Dire Dawa city administration and the Tigray region. The lowest, below 20% use of at least four ANC visits, were reported in the Somali and Afar regions. Despite the overall increase in ANC visits in the country, it was found that there was significant regional variation in undertaking four or more ANC visits.

Hot spots of at least four ANC visits were detected in the Southern Nations, Nationalities and Peoples region, and some parts of the Gambela and Oromia regions. The identified hot spots were located closest to teaching hospitals (Jimma, Hawassa, Wolaita and Arba Minch hospitals); they have a high number of service providers including students. These teaching hospitals also have antenatal care services available and are ready to serve the target population. Therefore, women who are living closest to these facilities are more likely to have frequent antenatal care visits, as the services could be more attractive to them.

The majority of cold spots were detected in the Addis Ababa city administration followed by some parts of the Oromia region. This was an unexpected finding, as Addis Ababa is where the majority of health facilities are concentrated. This highlights the need to specify how hot spot analysis works. In hot spot analysis, every feature has a neighborhood and that neighborhood is compared to the study area, and the feature is marked with the result of that comparison. If the neighborhood is significantly different from the study area, then that feature will be marked either a hot spot or a cold spot depending on whether there are high values or low values.

One important note here is that *‘where are the hot spots?’* is not necessarily the same as *‘where are the highest values?’* Hot spot analysis is a test of spatial randomness. In hot spot analysis, one can get a feature of low values, even zero, which is marked as a hot spot because its neighborhood was high enough to bring that local average to be significantly different from the global average. In our study finding, even though the overall prevalence of antenatal care use was high in Addis Ababa, the majority of clusters in the city had very low values. When every neighborhood in the city was compared to the study area, the neighborhood values were significantly lower than the study area. Thus, the spatial statistics marked every feature in Addis Ababa as a cold spot that is the local average is significantly lower than the global average. Due to this low prevalence clusters, Addis Ababa did not show hot spots of ANC4+ visits. Similarly, most of the clusters in Addis Ababa had the least first ANC visits during the first trimester. This could also be explained by the spatial variations of timing of first ANC visits as observed from the Moran eigenvector spatially varying coefficient (M-SVC) regression model. In Addis Ababa, it was found that first ANC visits during the first trimester were not strong predictors of at least four ANC visits as observed in the Amhara and Tigray regions. Even though the statistics gave this finding, further study is required to understand why low rates of at least four ANC visits are clustered in Addis Ababa.

Spatial regression models assume the potential between-neighborhood correlations due to spatial process [44]. Standard multilevel models, however, do not assume spatial dependence; neighborhood observations are independent of one another [44, 45]. This could lead to the overstatement of statistical significance of neighborhood effects [44]. Our paper considered same variables for both spatial and multilevel analysis. However, only wealth quintiles and availability of ANC supplements were shared between the two. This doesn’t mean that they have the same interpretation on ANC visit. Spatial regression models enables to identify what is happening in a particular geographic location and why that is happening. In the spatial regression analysis, it was found that four or more ANC (ANC4+) visits were varied across geographic areas. These geographical variations were explained by different variables, such as first ANC visits, availability of ANC supplements, facilities readiness to provide skilled care and distance to ANC providing facilities. Furthermore, it helped us to explain cold spots of ANC4+ visits in Addis Ababa of which the standard multilevel analysis could not explain it. These enables for informed decision making like which communities and health facilities need especial attention and where should the government spend more money.

Different individual and regional level factors were significantly associated with the use of more ANC visits (ANC4+ visits). Amongst the regional level variables, it was found that a one-unit increase in the mean score of antenatal care service availability in a typical region was significantly associated with a five-fold increase in the odds of having more ANC visits. In Nigeria, it was found that health facilities staffed with fewer antenatal care providers were negatively associated with the use of antenatal care services [23]. Availability and provision of antenatal care commodities at every antenatal care facility will help to reduce the costs associated with purchasing those drugs and thus improve women’s antenatal care visits. Furthermore, health facilities readiness, for instance, having the required number of physicians or services providers attending pregnant women would minimize the waiting time that a woman could spend at a health facility. This could make the service attractive and hence, get more women for antenatal care services very easily.

Every one-kilometre increase in distance to the nearest ANC providing facilities in a typical region was negatively associated with the odds of having more ANC visits. This finding was supported by a study carried out in Ethiopia where proximity to a health facility [21] was significantly associated with the use of antenatal care service. Furthermore, a study carried out in Northern India found that living far from a health facility was negatively associated with maternal health service use [17]. Similarly, distant health facilities were negatively associated with the use of antenatal care services [23, 24]. However, those women who were living close to obstetric health facility were more likely to use antenatal care services [46]. In Ethiopia, having access to obstetric care facilities within an-hour travel time was also significantly associated with the use of antenatal care services [47, 48]. In rural Burkina Faso, pregnant women who had access to obstetric care facility within 5 km was significantly associated with the odds of having at least three antenatal care visits [49]. This indicates that geographic accessibility, measured in either distance or travel time, has a greater influence on maternal health service utilization [50].

Similarly, pregnant women who were living in rural areas were 47% less likely to have at least four ANC visits as compared to urban women. This finding was supported by another study carried out in Ethiopia where place of residence as well as administrative region were significantly associated with antenatal care use [51, 52]. Furthermore, this was in agreement with previous studies carried out in Ghana [19], Vietnam [15], Nigeria [16] and Northern India [17]. Worldwide, the inequalities in the distribution of health facilities were reflected by the higher proportion of antenatal care use in urban centres as compared to rural areas [18, 53]. These disparities could be due to the local inaccessibility of obstetric care services in rural areas as well as variations in some regional administrations. Therefore, the government and other service providers should work together for improving communities’ easy access to healthcare services.

Amongst the individual-level factors, women’s autonomy in their own healthcare decision was significantly associated with the odds of having more ANC visits. A woman whose husband/partner made decisions on her own healthcare was 24% less likely to have at least four ANC visits as compared to a woman who had autonomy to make decisions. This was consistent with other study findings where husbands’ approval had a greater effect on the use of antenatal care services [48, 54]. Another study conducted in Ethiopia found that women’s autonomy on their own healthcare decision-making was significantly associated with the higher odds of using antenatal care service [52]. Therefore, the empowerment and autonomy of women in all aspects of life, especially in their own healthcare decision is a highly important end in itself.

In Ethiopia, among the individual-level variables, husbands’ level of education was significantly associated with the odds of having more ANC visits. This study found that a woman whose husband had attained a primary level of education was 53% more likely to have at least four ANC visits as compared to those whose husband had no education. This finding adds to previous research conducted in Ethiopia which found that women’s education [21, 22] and husband’s attitude [22] were significantly associated with antenatal care use.

The odds of having at least four ANC visits was significantly associated with the increased in household wealth, in agreement with previous research conducted in Ghana [19] and Nigeria [24]. In some settings, service fees and socio-economic status were strong predictors of antenatal care use [19, 20, 55]. In Ethiopia, even though antenatal services are free of charge at government health facilities, services fees at private health facilities as well as transportation costs are high. Moreover, most women end up spending the entire day at health facilities for their check-ups and on travelling to and from health facilities. This kind of indirect cost is associated with the women’s daily life of which they might go to a farm or market to make their daily living. Therefore, women in the highest wealth quintile will be more likely to make more antenatal care visits as compared to women in the lowest quintile.

In Ethiopia, having unwanted pregnancy was negatively associated with the likelihood of having more ANC visits as compared to wanted pregnancies. This finding was consistent with other studies carried out in Ethiopia, as those women who had a wanted pregnancy were more likely to have antenatal care visits [48, 52, 56]. Women with wanted pregnancies could want to have a healthy pregnancy and childbirth, and thus they might give great attention for antenatal care services.

In this current study, it was found that a one-child increase in the number of living children a woman had was significantly associated with a 7 % decrease in the likelihood of having more ANC visits. This finding was supported by studies carried out in Ghana [19] and India [57] where a significant reduction in the use of ANC services was observed with increasing in the number of living children. This could be related to woman’s previous experience, as a woman might be reluctant to have ANC visits in a subsequent pregnancy if she had a negative previous experience or if she perceived the importance of ANC to be low with subsequent pregnancies. To avoid any complications and/or adverse pregnancy outcomes, more attention should be given to encouraging women to have more ANC visits. However, in another study, it was found that high parity was significantly associated with higher uptake of ANC visits [52]. This could be attributable to previous complications and/or adverse pregnancy outcomes. Furthermore, this could be due to influences of previous ANC visits, in case if they had.

The identified individual and regional level factors such as distance from health facility, socio-economic status, number of living children and women’s autonomy might be related to each other. They do not exist as separate factors in life. For example, in a systematic review, it was found that poor geographic access to health care was overlapped with poverty. In Uganda, those regions with the worst access to health care were the regions where the large segment of the population lived below poverty line [58]. Distance was found to be a barrier in obtaining health care for 20% of the poorest as compared to only 9 % of the richest population in Uganda [59]. In low-and middle-income countries, it was found that improving healthcare access could reduce socio-economic gaps in healthcare [60].

This study linked population and health facility data to identify both the demand and supply side determinants of antenatal care use. This was not the case in most previous studies where they assessed the demand and supply side determinants separately. In addition to the standard multilevel analysis, this study identified geographical variations of ANC use as well as factors associated with these variations. Investigating ANC use geographically is very important for informed decision making and monitoring and evaluation purposes.

Even though this study had several methodological limitations, most of these were minimized. Problems related to sampled facilities, temporal differences between DHS and SPA surveys, and misclassifications errors were minimized [32]. However, using a straight-line distance introduces bias and this would be reduced if a road network link was carried out. In case of road network analysis, the distance between DHS clusters and health facilities would not be affected by terrain characteristics. It reflects the road distance rather than the shortest distance between two points. Furthermore, analysis of sampled facilities and removing DHS clusters without geographic coordinate information might under or overestimate the study finding. For instance, the influence of distance on ANC use could be different if all health facilities were included. The estimated average straight-line distance to the nearest ANC facility would not be this much high.

Even though multilevel analysis should include weights at each level, this study did not considered sampling weights. The problem with this is that DHS does not provide separate weights for different levels, such as region, cluster or household-level weights. DHS only provides an average weight which is proportional to *hv005 or v005*. The GLIMMIX procedure, however, asks each level weight. It has *OBSWEIGHT = option* and *WEIGHT = option*. The GLIMMIX procedure does not provide any other solution when we have average weights.

## Conclusion

In Ethiopia, even though there is an increase in the use of ANC services, the country has still not achieved the recommended number of ANC visits. It was found that husband’s/partner’s education, women’s autonomy in their own healthcare decision making, rural residence, ANC service availability and average distance to the nearest ANC providing facility were associated with having more ANC visits. There was a five-fold increase in the odds of having more ANC visits when key ANC supplements were available. Furthermore, there is evidence of a wide geographical variation in having at least four ANC visits across the country. Hot spots of at least four ANC visits were identified in areas where there are teaching hospitals. The findings of this study have several implications: first, beyond providing free antenatal services at public health facilities, the government and non-governmental organizations should make an effort to set up health facilities in rural areas to improve ANC use. Second, availing ANC services at all levels, especially in rural areas and some regions with poor healthcare access, and making them ready to provide these services should also be prioritized. In addition to this, the available and newly constructed teaching hospitals should be equipped to provide ANC services. Third, the empowerment and autonomy of women in all aspects of life, especially in their own healthcare decision, need to be emphasized. Lastly, as observed from the hot spot analysis, there is a need for gaining a more detailed understanding of the findings for Addis Ababa.

## Data Availability

Not applicable.

## Abbreviations

ANC: Antenatal Care
ANC4 +: Four or more Antenatal Care Visits
CRS: Coordinate Reference System
DHS: Demographic and Health Survey
EAs: Enumeration Areas
EDHS: Ethiopia Demographic and Health Survey
ESPA+: Ethiopia Service Provision Assessment Plus
FDR: False Discovery Rate
GIS: Geographic Information Systems
HGLM: Hierarchical Generalized Linear Model
ICC: Intra-class Correlation Coefficient
SNNPR: Southern Nations, Nationalities and Peoples Region
SPA: Service Provision Assessment
TFR: Total Fertility Rate
WGS84: World Geodetic System 84
WHO: World Health Organization
